# Designing xylan for improved sustainable biofuel production

**DOI:** 10.1111/pbi.13150

**Published:** 2019-09-30

**Authors:** Dyoni M. Oliveira, Thatiane R. Mota, Fábio V. Salatta, Rogério Marchiosi, Leonardo D. Gomez, Simon J. McQueen‐Mason, Osvaldo Ferrarese‐Filho, Wanderley D. dos Santos

**Affiliations:** ^1^ Laboratory of Plant Biochemistry Department of Biochemistry State University of Maringá Maringá Paraná Brazil; ^2^ Centre for Novel Agricultural Products Department of Biology University of York York UK

**Keywords:** arabinoxylan, bioenergy, ferulic acid, lignocellulosic biomass, plant cell wall, saccharification

## Background

Increasing greenhouse gas emissions and diminishing supplies of fossil‐derived fuels underline the need for environmentally sustainable energy resources. Lignocellulose, more generically named simply as plant biomass, represents one of the most abundant renewable resources for biofuels. Lignocellulosic biomass from forest residues, agro‐wastes and energy grasses is extensively exploited for bioenergy production. This renewable biomass source is abundant, highly accessible, relatively cheap and diversifies the energy matrix (Marriott *et al*., 2016). Grass lignocellulosic material consists mostly of secondary cell walls and is composed mainly of cellulose (25%–55%), hemicellulose xylan (20%–50%), lignin (10%–35%) and small amount of pectin, depending on plant species, organ, cell types and developmental stage of the tissue. Although pectin is a minor cell wall component, there is increasing evidence suggesting that pectic polysaccharides are involved in cell wall recalcitrance (Biswal *et al*., 2018).

**Figure 1:**
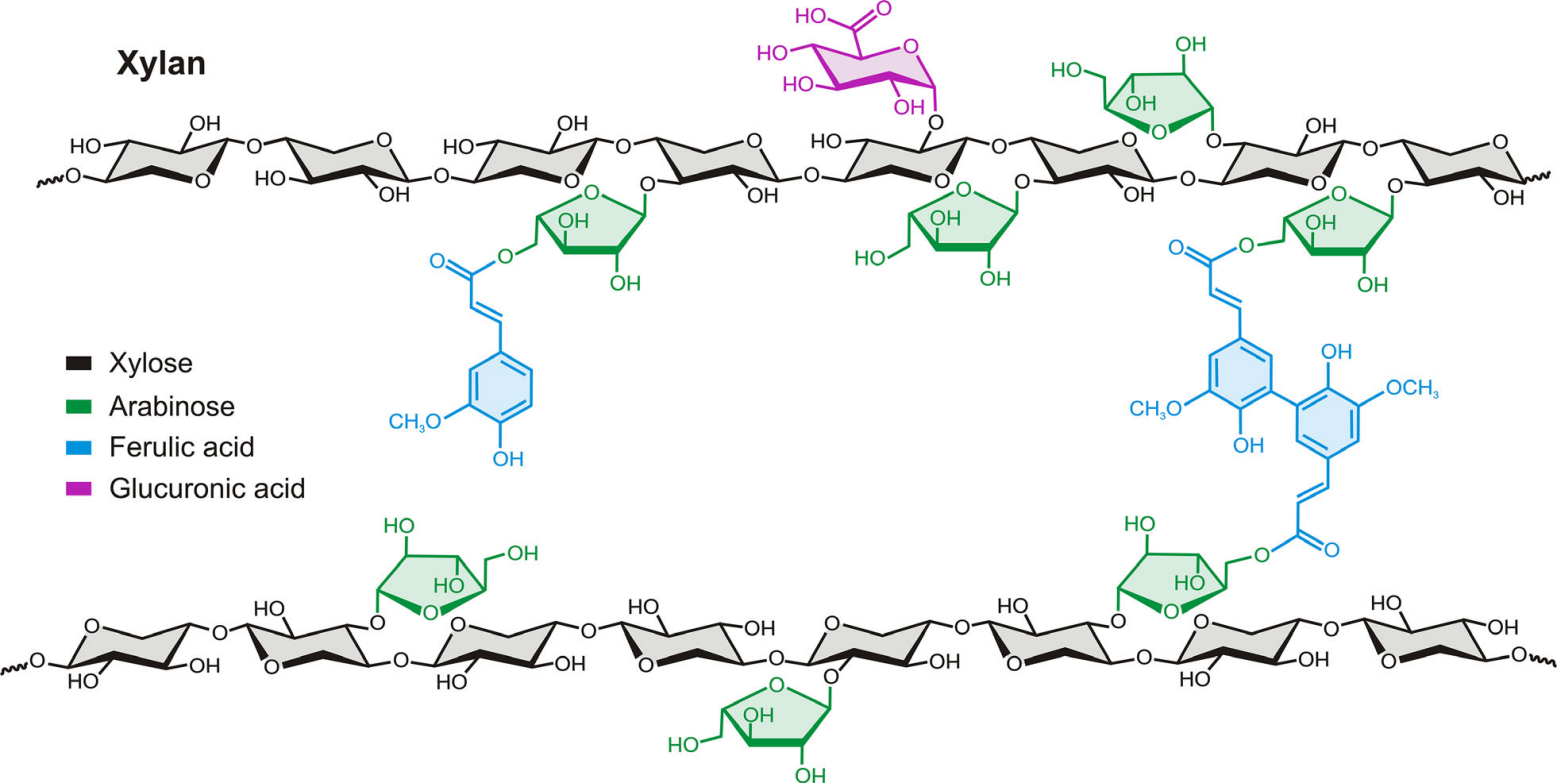
Generalized structure of xylan branched with arabinose, ferulic acid and glucuronic acid.

The most important crops farmed at large scale are grasses, where xylan is the main hemicellulose component in their cell walls. Xylan is tightly associated with cellulose microfibrils, connecting them through hydrogen bonds. Recently, it was demonstrated that xylan interacts with cellulose by twofold helical screw conformation and the interaction is influenced by xylan substitution patterns (Simmons *et al*., 2016). The xylan backbone consists of a linear chain of β‐(1,4)‐D‐xylosyl residues (Xyl*p*) and makes up between 20% and 35% of the total cell wall. Arabinofuranose residues (Ara*f*) may be α‐(1,2) or α‐(1,3) linked to the xylan backbone forming arabinoxylan (AX), which may be further substituted with ferulic (FA) or *p*‐coumaric acid residues (Figure 1). The side‐chain decorations on the AX backbone vary between plant species and tissues. In grasses, the primary and secondary cell walls contain substantial amounts of AX, which is also found at much lower abundance in primary cell walls of dicots. For more background information of xylan biosynthesis and modifications, see the review by Smith *et al*. (2017).

Ferulic esters in AX might undergo oxidative dimerization to form cross links at adjacent AX chains or lignin, thereby generating intramolecular and intermolecular cross links of AXs with lignin and structural proteins that contribute to the recalcitrance of grass biomass for saccharification. FA may act as a nucleating site for the formation of lignin, hence linking AXs to lignin by forming a lignin–AX complex (Oliveira *et al*., 2015). Recent studies of the lignin–polysaccharide interactions in secondary cell walls demonstrated that the hydroxyl groups in xylan have abundant electrostatic interactions with lignin methoxyl groups found mainly in S‐lignin (Kang *et al*., 2019).

Arabinoxylan also influences the enzymatic hydrolysis of cellulose, and it requires enzymes different from those used to hydrolyse cellulose. Lowering AX content and/or its decorations (with ara*f* and FA) reduces the cross‐linkages among AX, lignin and cellulose in plant cell walls, decreasing biomass recalcitrance (Smith *et al*., 2017). Research aiming at elucidating the genes required for xylan biosynthesis, the way they are controlled and how changes in these genes influence plant development has been boosted by the potential of plant biomass as a source of renewable energy. Recent studies have highlighted the key role that xylan plays in the conversion of lignocellulosic feedstocks to fuels and other value‐added products.

## Tailoring xylan structure

The glycosyltransferases (GTs) required for xylan biosynthesis were first identified in *Arabidopsis thaliana*. These enzymes catalyse the biosynthesis of the xylan backbone, transferring nucleotide sugars to the growing AX chain within the Golgi apparatus. Two members of the glycosyltransferase family 43 (GT43), IRREGULAR XYLEM9 (IRX9) and IRX14 proteins, and one member of GT47, IRX10, are implicated in the biosynthesis of the xylan backbone, but the specific role is not completely established (Brown *et al*., 2007; Smith *et al*., 2017). Subsequent works confirmed that IRX10 is the β‐1,4‐xylan xylosyl transferase responsible for xylan polymer extension, transferring xylosyl residues from UDP‐xylose to xylooligosaccharides at the reducing end, whereas IRX9 and IRX14 are accessory proteins involved in the elongation of the xylan backbone and are structural components of the functioning xylan synthase complex (XSC). Additionally, glucuronosyltransferases (GUX) from the GT8 family and arabinosyltransferases (XAT) from the GT61 family are responsible for the addition of glucuronosyl and arabinosyl on the xylan backbone, respectively (Smith *et al*., 2017).

Despite the importance of AX for biofuels, the biochemical function and structure of GT43 enzymes are still unclear. GTs are difficult to study because they are labile, present in multimeric complexes and encoded by large gene families whose members can have overlapping functions. Almost all the studies with the GT43 family are limited to comprehensive genetic analysis of the functional roles of GT43, mutations in the genes *IRX9* and *IRX14* result in decreased xylan synthase activity and xylose content, accompanied by a shorter xylan backbone (Brown *et al*., 2007).

Recent advances provided important evidence that *BdGT43A*, the orthologue of *IRX14* in *Arabidopsis*, is involved in xylan backbone biosynthesis in *Brachypodium distachyon* (Whitehead *et al*., 2018). Using commercial cellulases, *B. distachyon* recombinant inbred lines (RILs) were screened, associating them with a single quantitative trait locus (QTL) for saccharification. The study revealed that RNAi suppression of *BdGT43A* in *Brachypodium* decreases xylose and arabinose content and increases stem saccharification relative to the wild type, which is clear genetic evidence that *Bd*GT43A is involved in xylan biosynthesis. In addition, the transgenic lines showed a decrease in FA and an increase in *p‐*coumaric acid, compared to the wild type. Similarly, plants of hybrid aspen (*Populus tremula × tremuloides*) down‐regulated simultaneously for *PtGT43B* and *PtGT43C*, the orthologues of *IRX9* and *IRX14,* respectively, present reduced xylose content relative to the reducing end sequence in xylan, with slight alteration in the chemical composition of wood, a small decrease in S and H lignin, accompanied by a higher lignocellulose saccharification efficiency (Ratke *et al*., 2018).

It is interesting to note that the underlying mechanisms for the reduced recalcitrance in Brachypodium and hybrid aspen transgenic lines are quite different. In *BdGT43A* silenced lines, the lower xylan content associated with decreased FA content is the main factor responsible for the increased saccharification efficiency. Alternatively, the reduction in the xylan in hybrid aspen *GT43* suppressed lines is associated with a small decrease in S and H lignin content, being this the main contribution to the higher saccharification efficiency. Different responses to the suppression of orthologues genes are due in part to the largely unpredictable pleiotropic effects and phenotypes associated with the mutations. Additionally, the mechanistic relationship between *GT43* gene repression and cell wall modifications requires more investigation.

Further examination of AX feruloylation (de Souza *et al*., 2018) identified a member of the BAHD acyltransferase family involved in the transference of FA residues to the AX backbone. Silencing the *SvBAHD01* gene by RNAi in *Setaria viridis* reduced FA content by 60% and increased stem saccharification efficiency (from 40 to 60%), without changing biomass productivity. Therefore, the increase in stem saccharification obtained by Whitehead *et al*. (2018) reflects a synergic effect of the overall decrease in feruloylation of arabinosyl moieties linked to AXs. The elucidation of the genes involved in xylan biosynthesis and feruloylation, the way they are controlled and how changes in these genes influence plant growth can facilitate the design of strategies aimed at engineering plants to exhibit modified xylan for improved biofuel production. These recent insights emphasize the importance of generating plants with reduced FA and AX content in the search for improved feedstocks for biorefineries.

## Future perspectives

Arabinoxylans are abundant in nature and in the grass cell wall. AX and FA are essential components, cross‐linking polysaccharides to lignin and increasing the cell wall resistance to hydrolysis. Although further elucidation of xylan biosynthesis mechanisms is still necessary, a possible model to explain how it is associated with biomass digestibility is emerging. The advantage of discovering the genes associated with the expression of the enzymes in the biosynthesis of xylan is that there are now more ways to design this structure. Genetic manipulation of xylan biosynthesis and feruloylation raises many interesting questions that should be addressed in the future and is a potential approach to engineering crops that match the industrial requirements for food, cellulosic ethanol and biorefineries.

## Conflict of interest

The authors declare no competing interests.
